# Supplementary material for: Caloric restriction reprograms adipose tissues in rhesus monkeys
Source: bioRxiv. 2025 Mar 5:2025.03.03.641286. Preprint. [Version 1] doi: 10.1101/2025.03.03.641286 (PMC11908232; doi:10.1101/2025.03.03.641286)
Supplement: Supplement 1 [file NIHPP2025.03.03.641286v1-supplement-1.pdf]

bioRxiv preprint doi: https://doi.org/10.1101/2025.03.03.641286; this version posted March 5, 2025. The copyright holder for this preprint (which was not certified by peer review) is the author/funder, who has granted bioRxiv a license to display the preprint in perpetuity. It is made available under aCC-BY 4.0 International license.

Supplementary Figure S1: Blood analyses and biometric data for individuals on study.
(**A**) Graphs showing biometric and blood chemistry measures for individuals on the Control or CR diet (n=4; bar, mean; line, SD). Significance indicated by * ($p<0.05$) or p-value listed from student t-test. Measures include: age, weight, fat, lean, appendicular lean mass, total and total abdominal fat percent, HOMA-IR, glycosylated-hemoglobin (Glyc HGB), basal glucose and insulin, insulin sensitivity (Si), cholesterol, triglycerides, white and red blood cell counts (WBC and RBC), hemoglobin (Hgb), hematocrit (Hct), blood urea nitrogen (BUN), creatinine, lactate dehydrogenase (LDH), gamma-glutamyl transferase (GGT), alanine aminotransferase (ALT), aspartate aminotransferase (AST), alkaline phosphatase (ALP), total protein, albumin, total bilirubin.

Supplementary Figure S2: Depot-specific expression of Adipokines and SenNet factors.
Heatmaps of SAT/VAT Log2FC for Adipokines (left) and SenNet factors (right). Filled dot denotes passing adjusted $p<0.05$, empty dot denotes unadjusted $p<0.05$. Related to Figure 1.

Supplementary Figure S3: Depot-specific transcriptional response to CR.
(**A**) Bar chart depicting the Log2FC (CR/C) of DEGs that are shared or unique to the three comparisons: both depots (gray), SAT (yellow) or VAT (blue). (**B**) Principal component analysis plot of the transcriptome for each group (n=4). Ellipse denotes 80% confidence level. Related to Figure 2.

Supplementary Figure S4: WGCNA module-trait associations.
Correlation of module eigengenes with individual biometric measures (traits). Boxes contain module-trait correlations (top) and pvalue (bottom). Numbers of genes per module are listed on the right. Related to Figure 4.

Supplementary Figure S5: Blue module trait associations enrich for oxidative phosphorylation gene containing pathways.

bioRxiv preprint doi: https://doi.org/10.1101/2025.03.03.641286; this version posted March 5, 2025. The copyright holder for this preprint (which was not certified by peer review) is the author/funder, who has granted bioRxiv a license to display the preprint in perpetuity. It is made available under aCC-BY 4.0 International license.

(**A**) Scatter plots of gene significance versus blue module-membership for genes associated with triglycerides, lean and appendicular mass, and insulin sensitivity (Si). n (number of genes), cor (correlation), p (pvalue); (**B**) Bar plot of KEGG pathways enriched via ORA (qvalue<0.05) for significant blue module genes (gene significance p<0.05) for each trait association. Related to Figure 5.

bioRxiv preprint doi: https://doi.org/10.1101/2025.03.03.641286; this version posted March 5, 2025. The copyright holder for this preprint (which was not certified by peer review) is the author/funder, who has granted bioRxiv a license to display the preprint in perpetuity. It is made available under aCC-BY 4.0 International license.

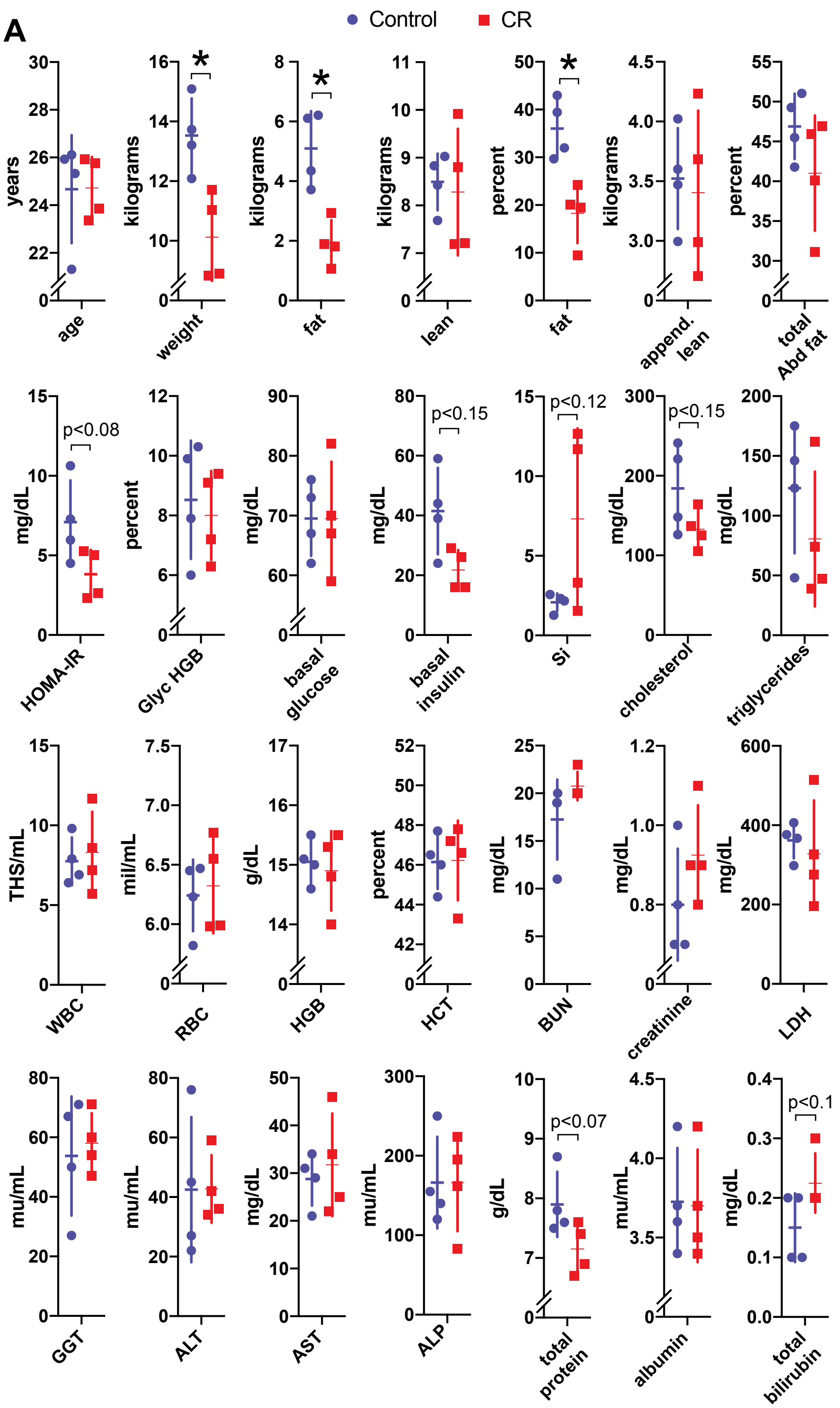

Supplementary Figure S1

bioRxiv preprint doi: https://doi.org/10.1101/2025.03.03.641286; this version posted March 5, 2025. The copyright holder for this preprint (which was not certified by peer review) is the author/funder, who has granted bioRxiv a license to display the preprint in perpetuity. It is made available under aCC-BY 4.0 International license.

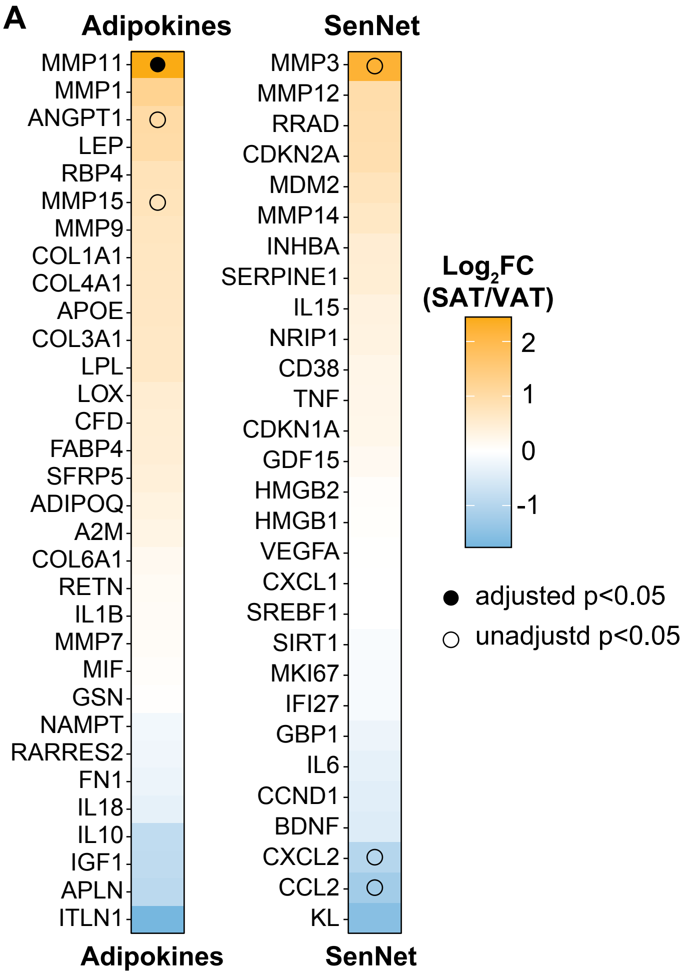

Supplementary Figure S2 (Related to Figure 1)

bioRxiv preprint doi: https://doi.org/10.1101/2025.03.03.641286; this version posted March 5, 2025. The copyright holder for this preprint (which was not certified by peer review) is the author/funder, who has granted bioRxiv a license to display the preprint in perpetuity. It is made available under aCC-BY 4.0 International license.

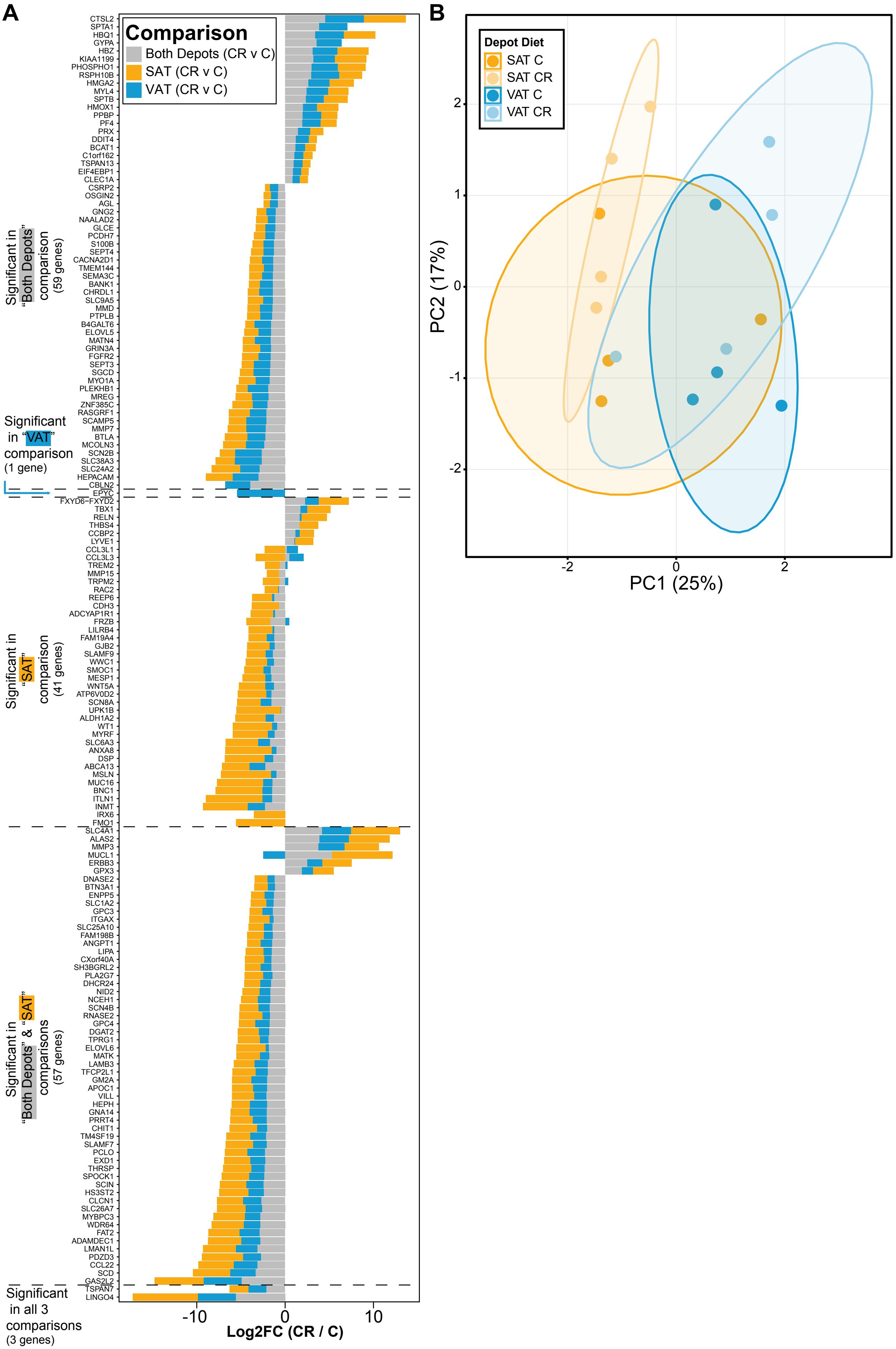

Supplementary Figure S3 (Related to Figure 2)

bioRxiv preprint doi: https://doi.org/10.1101/2025.03.03.641286; this version posted March 5, 2025. The copyright holder for this preprint (which was not certified by peer review) is the author/funder, who has granted bioRxiv a license to display the preprint in perpetuity. It is made available under aCC-BY 4.0 International license.

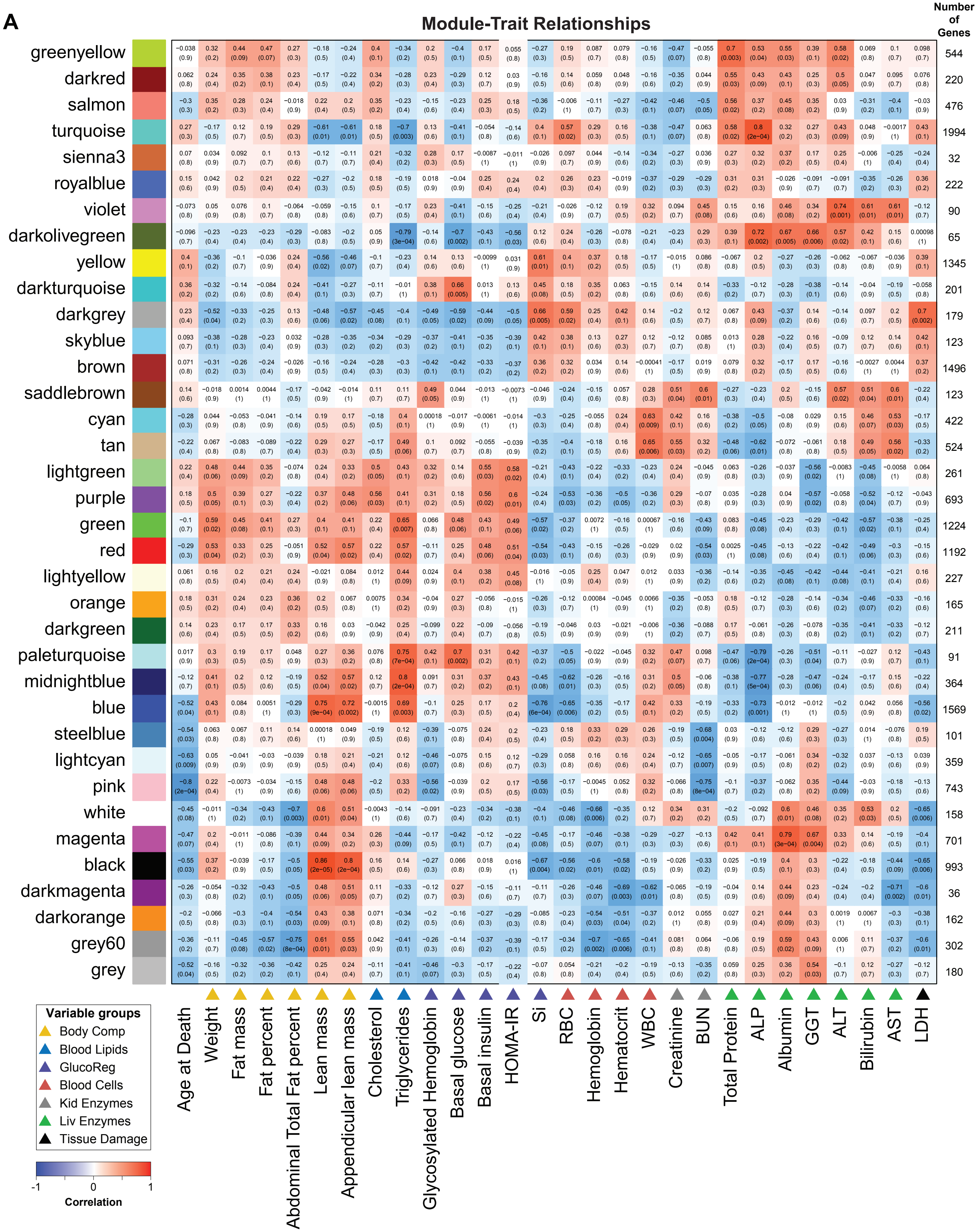

Supplementary Figure S4 (Related to Figure 4)

bioRxiv preprint doi: https://doi.org/10.1101/2025.03.03.641286; this version posted March 5, 2025. The copyright holder for this preprint (which was not certified by peer review) is the author/funder, who has granted bioRxiv a license to display the preprint in perpetuity. It is made available under aCC-BY 4.0 International license.

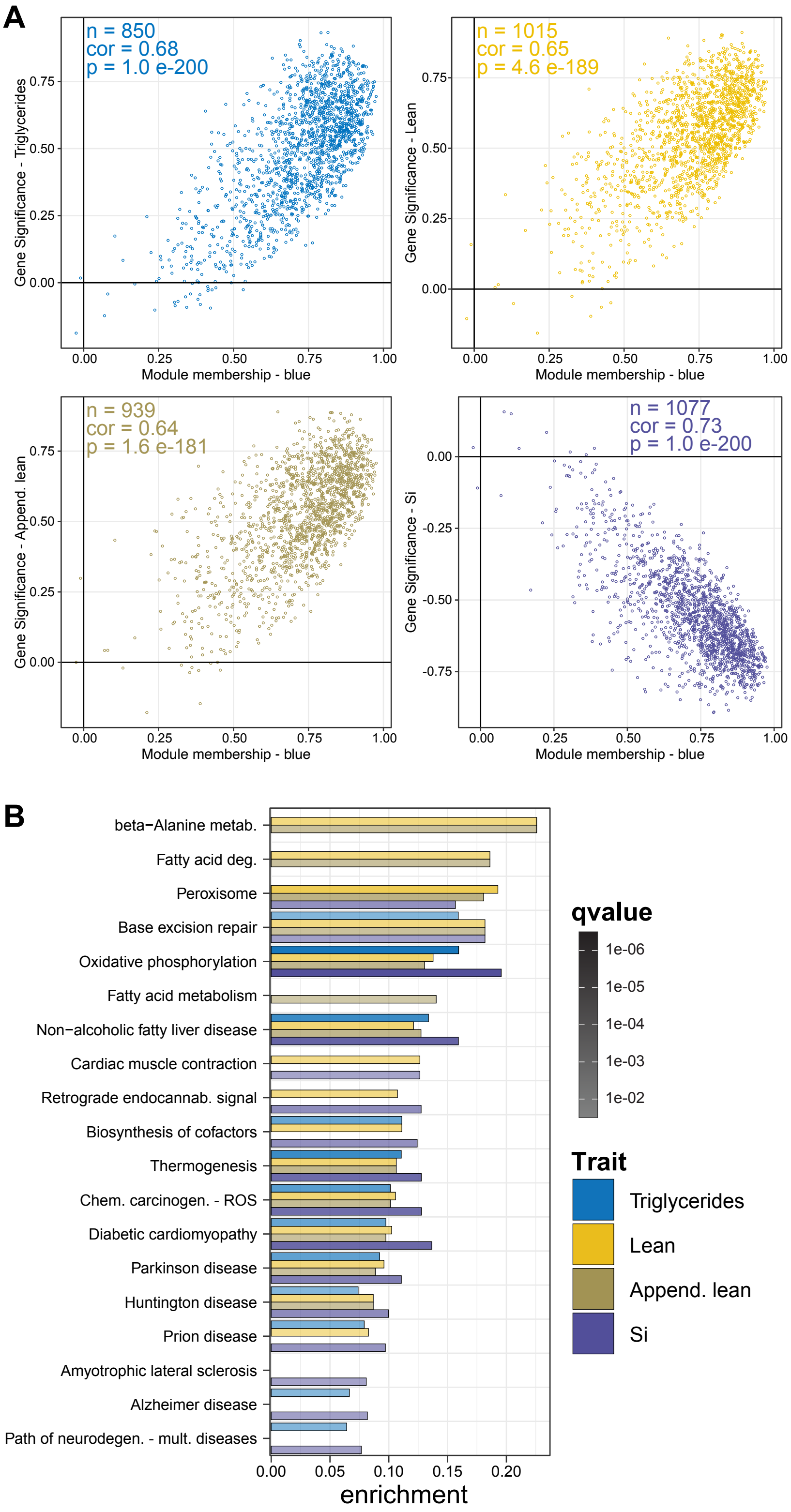

Supplementary Figure S5 (Related to Figure 4)
